# Artificial Proprioceptive Feedback for Myoelectric Control

**DOI:** 10.1109/TNSRE.2014.2355856

**Published:** 2014-09-09

**Authors:** Tobias Pistohl, Deepak Joshi, Gowrishankar Ganesh, Andrew Jackson, Kianoush Nazarpour

**Affiliations:** Institute of Neuroscience, Newcastle University, UK. He is now with Bernstein Center Freiburg, University of Freiburg; Department of Electrical and Electronics Engineering, Graphic Era University, Dehradun - 248002, India; CNRS-AIST JRL (Joint Robotics Laboratory), UMI3218/CRT, Intelligent Systems Research Institute, Tsukuba, Japan-305-8568 and the Centre for Information and Neural Networks (CINET-NICT), Osaka, Japan-5650871; Institute of Neuroscience, Newcastle University, Newcastle upon Tyne, NE2 4HH, UK; School of Electrical and Electronic Engineering and the Institute of Neuroscience, Newcastle University

**Keywords:** Electromyogram signal, proprioceptive feedback, sensorimotor integration

## Abstract

The typical control of myoelectric interfaces, whether in laboratory settings or real-life prosthetic applications, largely relies on visual feedback because proprioceptive signals from the controlling muscles are either not available or very noisy. We conducted a set of experiments to test whether artificial proprioceptive feedback, delivered non-invasively to another limb, can improve control of a two-dimensional myoelectrically-controlled computer interface. In these experiments, participants’ were required to reach a target with a visual cursor that was controlled by electromyogram signals recorded from muscles of the *left* hand, while they were provided with an additional proprioceptive feedback on their *right* arm by moving it with a robotic manipulandum. Provision of additional artificial proprioceptive feedback improved the angular accuracy of their movements when compared to using visual feedback alone but did *not* increase the overall accuracy quantified with the average distance between the cursor and the target. The advantages conferred by proprioception were present only when the proprioceptive feedback had similar orientation to the visual feedback in the task space and not when it was mirrored, demonstrating the importance of congruency in feedback modalities for multi-sensory integration. Our results reveal the ability of the human motor system to learn new inter-limb sensory-motor associations; the motor system can utilize task-related sensory feedback, even when it is available on a limb distinct from the one being actuated. In addition, the proposed task structure provides a flexible test paradigm by which the effectiveness of various sensory feedback and multi-sensory integration for myoelectric prosthesis control can be evaluated.

## I Introduction

Myoelectric interfaces use the electrical activity of muscles (electromyogram (EMG)) to control computers or electrically actuated devices, such as prosthetic limbs [1]. During the operation of myoelectric interfaces the user typically relies on visual information as the main source of feedback about the state of the device. While there have been several attempts to deliver sensory feedback about the state of the interface through grip force feedback via vibro-tactile stimulation [2], vibro-tactile, mechano-tactile or electro-tactile stimulation [3]–[5], and feedback of the prosthetic joint angle or position through cutaneous stimuli [7],[8], it is not yet clear whether provision of other sensory signals in addition to vision would augment control of myoelectric interfaces. This is because, conventionally, the effectiveness of these sensory signals is quantified when vision is withheld. The aim of this paper is to develop a simple paradigm by which 1) the usefulness of the added feedback modality delivered to an intact body organ can be examined in different visual feedback conditions and 2) the importance of the congruency between different feedback modalities can be quantified.

Only limited attention has been given to the provision of positional cues as feedback via proprioception; that is “the perception of joint and body movement as well as position of the body, or body segments, in space” [9]. Proprioceptive feedback provided mechanically to the arm using an exoskeleton has been shown to improve monkeys’ performance in a brain-machine interface task [10]. In a study with able-bodied humans [11], subjects controlled the motion of a virtual finger to grasp a virtual object via a grasping force input measured at the thumb. The grasping force controlled proprioceptive feedback that was felt at the index finger. It was shown that additional proprioceptive feedback could improve control of a visual representation of the grasp, albeit only for small target sizes.

Recently in [12], it was shown that the use of proprioceptive feedback via an exoskeleton in a non-invasive brain computer interface experiment can enhance sensorimotor de-synchronization of the brain rhythms. However, in myoelectric interfaces (e.g. myoelectric prostheses), traumatic event of amputation can impair their peripheral sensorimotor connections at the site of injury. Hence, delivering biologically-accurate proprioceptive feedback through the controlling limb for amputees have only been possible using invasive electrodes [13] and sophisticated electronics [14] positioned at more proximal sites.

Here we examined an alternative strategy, proprioception delivery on a limb different than that controls a myoelectric task, and investigated whether sensorimotor association can be learnt in such a strategy. A similar concept was previously employed in a simple one-dimensional task by Wheeler et al [8]. However, since provision of artificial proprioception to the controlling limb was not their primary purpose, they did not evaluate its usefulness in absence or presence of the visual feedback.

We developed a myoelectric interface task in which the participants controlled the position of a computer cursor by myoelectric activity associated with small isometric contractions of the muscles in their *left* hand and arm. Subjects received artificial proprioceptive feedback (PF) to their *right* arm that was moved passively by a robotic manipulandum.

The interaction between different sensory modalities has long been a subject of research [15], [16]. In contrast to visual information that is encoded in the extrinsic coordinates, proprioceptive information is encoded in a body-centered coordinate system [17], [18]. Therefore, for artificial proprioceptive feedback to be useful in a visually instructed task - such as the proposed setup - the proprioceptive information must be transformed and integrated with the visual information. We examined whether such multi-sensory integration across limbs can be learnt and the extent to which this depends on the spatial congruency between the proprioceptive and visual feedback.

In section II, we present the hardware for delivery of the artificial proprioception, the methods of recording and analysis of the EMG signals and finally the details of our three experimental protocols. Results of the experiments are reported in Section III, before we discuss their significance and conclude in Section IV.

## II Method

### A Subjects

40 healthy right-handed subjects took part in three experiments: 21 in Experiment 1 and 19 in both Experiments 2 and 3. The latter two were run in close succession. All subjects gave their informed written consent before participation. The study was approved by the local ethics committee at Newcastle University.

### B Artificial proprioception

**Fig. 1:**
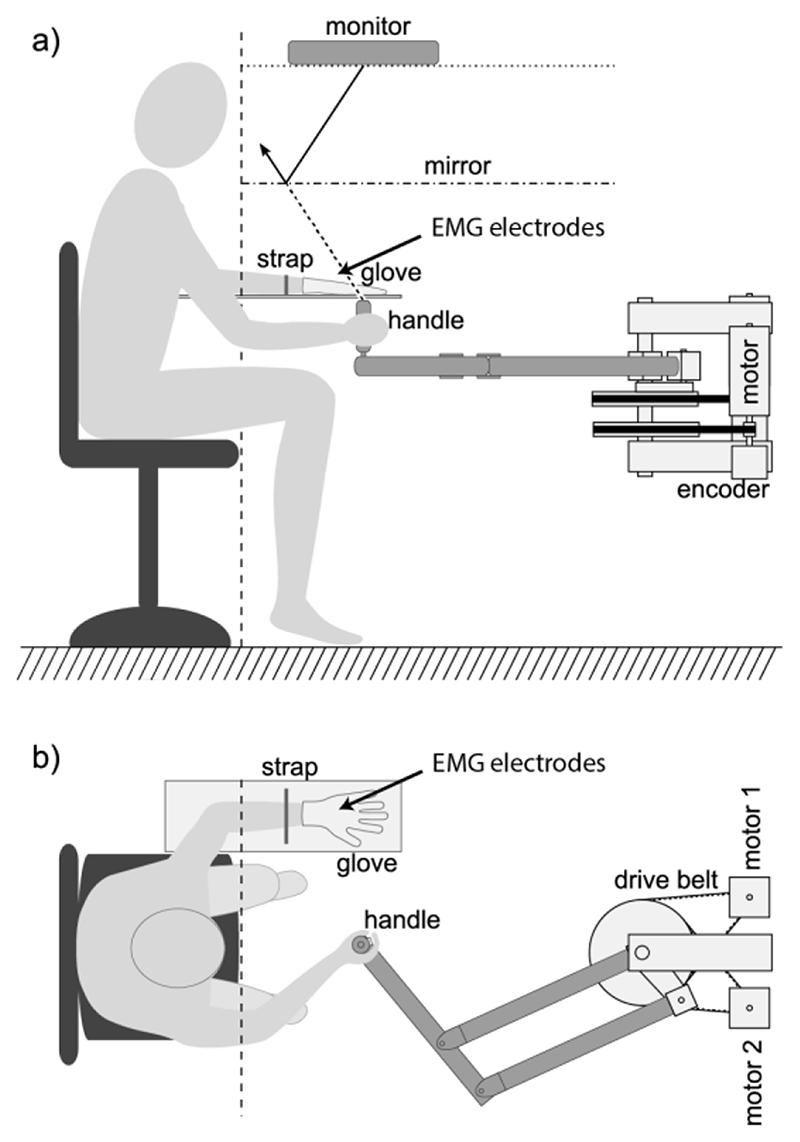
Schematic view of the experimental setup. (a) Side view: motorized manipulandum guiding the non-controlling, right hand along the cursor trajectory. Cursor and targets were projected from a monitor mirror system such that the visual cursor appears in the same plane as the handle of the manipulandum. (b) Top view: subjects controlled the task through isometric contractions in their left hand, immobilized on a horizontal arm rest, while the right hand was guided in the horizontal plane, congruent with cursor position.

We used an active manipulandum to provide artificial proprioceptive feedback about the cursor to subjects’ right arms, by guiding their hands along a movement trajectory that was controlled by myoelectric activity measured on their other, left hand and arm. A schematic view of the setup can be seen in Fig. 1. The robotic device was constructed in-house, similar to the vBot setup described in [19]. It consisted of a parallelogram arm, powered by two motors via drive belts that adjusted the angles of the two arm links. Angular positions were monitored through incremental encoders on each drive axis. A rotating handle was mounted onto the end of the arm, housing a button that had to be pushed with the index finger while holding the handle in order to supply power to the motors.

We immobilized subjects’ controlling left hands and arms on an armrest with a modified glove and a Velcro strap. The glove was glued to a board that was mounted on the armrest (Fig. 1). The armrest was mounted high enough to allow for unobstructed movement of the handle. Subjects observed the contents of a computer monitor, mounted on top, through a semi-transparent mirror so that they perceived a virtual horizontal display at the same height as the tip of the handle they were holding (Fig. 1a). During experiments, lights were switched off so that subjects did not receive visual feedback of their arm and could view only the computer display.

In some experimental conditions, we made the manipulandum closely follow a visual cursor moving in the virtual plane using a standard proportional-integral-derivative (PID) controller, with the cursor visible at the position of the handle. The coefficients of the PID controller were first determined using the Ziegler–Nichols method and then fine-tuned manually to avoid strong vibrations in the motion feedback which could perturb proprioception by introducing sensory noise. To this end, we optimized the PID controller to track only low-frequency movements that were relevant to the task by reducing control stiffness. PID control was fine-tuned to achieve a trade-off between accuracy of tracking and smoothness of movements; since fast, low-amplitude variability within the cursor position could superimpose a vibratory movement component onto the overall trajectory of the manipulandum and deteriorate proprioceptive feedback. We sought to avoid this by empirically decreasing the integral gain of the PID controller to allow smoother trajectories at the expense of sacrificing some level of stiffness and accuracy and the match between movements of cursor and manipulandum. Table I lists correlation coefficients, temporal lags and average distance between cursor and handle positions.

Importantly, movement of the handle did not influence cursor position so that the experimental task could not be affected at all by subjects’ right arm movements. Nevertheless, subjects were strongly discouraged from moving or resisting the manipulandum actively.

### C Electromyography Recordings

**Fig. 2:**
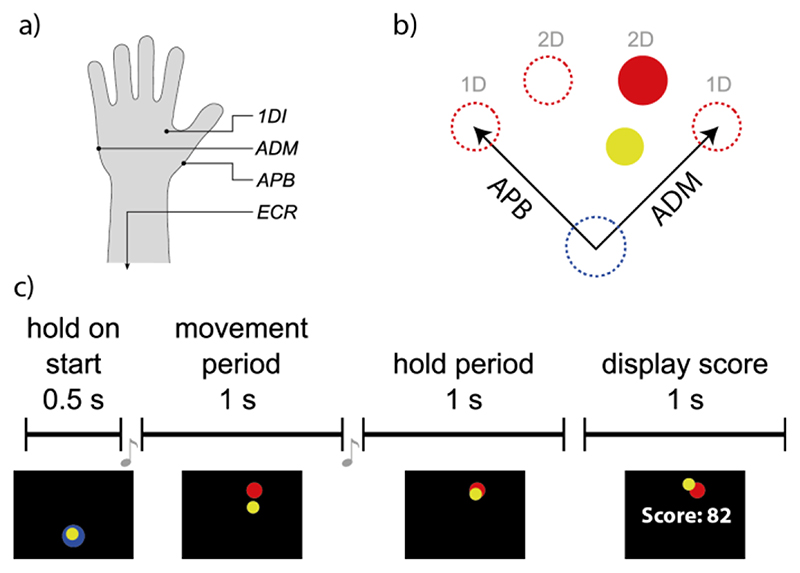
Experimental layout. (a) EMGs were recorded from muscles in the left hand and forearm; (b) task structure; (c) Trial structure. Subjects performed a virtual 4-target center-out task by controlling a cursor (yellow) from a starting zone (blue) to one of four target positions (red) with activations of two different muscles (Experiments 1 and 2: APB and ADM; Experiment 3: 1DI and ECR). Lateral targets required only one-dimensional control while the two central targets could only be reached with contributions from both muscles.

We recorded the EMG signal from the muscles of the left hand and forearm as indicated in Fig. 2a. For Experiment 1, the EMGs were recorded from abductor pollicis brevis (APB) and abductor digiti minimi (ADM). For Experiments 2 and 3, we recorded additional signals from the first dorsal interosseus (1DI) and extensor carpi radialis (ECR) muscles. APB, ADM and 1DI are intrinsic hand muscles, abducting thumb, little finger and index finger, respectively; ECR is located in the forearm and extends the hand at wrist level. Adhesive gel electrodes (Bio-logic, Natus Medical Inc., USA) were positioned over the belly of the muscle and an adjacent knuckle in the case of the intrinsic hand muscles, or on two positions along the muscle in the case of ECR. Myoelectric signals were amplified by a NeuroLog system (NL844/NL820A, Digitimer, UK) with the gain adjusted between 100 and 5000, band-pass filtered between 30 Hz and 1 kHz and subsequently digitized and transmitted to a PC at 2500 samples per second (NI USB-6229 BNC, National Instruments, USA). A Python-based graphical user interface was developed to implement data acquisition.

Before the start of an experiment, we recorded signal offset as well as amplitude of the measured signal, during rest and during comfortable contraction, for each EMG channel separately. To determine comfortable contraction levels, subjects were instructed to contract each muscle at a level that could be comfortably maintain and repeat many times without fatigue. In our previous studies with similar myoelectric interfaces [20]–[22], this level corresponded to an activity between 10-20% of the maximum voluntary contraction. Any encountered signal offset was subtracted from each channel as the first pre-processing stage. Instantaneous activation levels of recorded muscles *y* were estimated by smoothing (with a rectangular window) the preceding 750 ms of rectified EMG both during online processing and the assessment of activation levels of calibration data. This smoothing procedure slows the movement of the cursor, however probably due to this continuous update and the relatively slow movement in our task we found that (also in the previous work of Radhakrishnan et al (2008) [21] where in fact the smoothing window was 800ms in one experimental condition), subjects could adapt to it quickly. The subjects did not report the delay to impede their task.

The same procedure was repeated to calculate the rest *y_r_* and the comfortable contraction *y_c_* levels.

During the experiments, a normalized muscle activation level *y_n_* was computed for every channel independently by dividing the instantaneous level y by the level of comfortable contraction *y_c_* after resting levels *y_r_* were subtracted from either:

(1) $$y_{n}=\frac{y-y_{r}}{y_{c}-y_{r}}$$

### D Experimental protocols

The experiment consisted of a myoelectric-controlled center-out task with four circular targets (⌀2.4 cm) at 45°, 75°, 105° and 135° on a quarter circle of 8.6 cm radius around the circular starting zone (⌀3.6 cm) in the lower part of the workspace (Fig. 2b). The position of a yellow cursor (⌀1.8 cm) was determined by the activation levels of the two controlling muscles. The subjects controlled the position of the cursor such that contraction of a muscle caused the cursor to move along the muscle’s direction of action (DoA) proportional to the online estimated normalized muscle activation level, whereas relaxation brought the cursor back to the starting position (see equation 2). The two DoA vectors were pointing out from the starting point in 45° and 135° direction, as shown in Fig. 2b. The arrangement of DoAs was designed to be unintuitive, that is, DoAs were not reflected in movements the respective muscle would cause in the hand during natural movement. We deliberately avoided intuitive DoAs in our experiments to slow down the learning process and better observe improvements and sensorimotor integration over time.

The four target positions were divided into two groups: 1D targets, represented by the lateral positions (45° and 135°), and 2D targets which included the two central targets (75° and 105°). For movements to 1D targets, activation of the muscle with a DoA perpendicular to target direction was ignored, which resulted in a simpler, one-dimensional control scheme. For 2D targets, two-dimensional cursor position **x** was determined by the vector sum of both DoA vectors, scaled by the normalized activation level y_n_ of their respective muscle:

(2) $$\text{x}=\overset{2}{\underset{i=1}{\sum^{\;}}}y_{n,i}DoA_{i}.$$

Each trial consisted of four distinct phases, outlined in Fig. 2c. At the beginning a blue circle in the lower work-space indicated the starting zone. The experiment continued only after the yellow cursor was held continuously within the starting zone for 0.5 s. An auditory signal (250 ms long at 660 Hz) marked the beginning of a movement period during which one of the four targets was shown instead of the starting zone. During this period of 1 s, subjects were asked to move the cursor to the newly presented target and try to maintain the cursor inside the target during the ensuing hold period, marked by another auditory cue (250 ms long at 880 Hz), for one more second. A performance related score was calculated and presented to the participants at the end of each trial. The score reflected the percentage of time the cursor overlapped, even partially, with the target circle during the hold period. To calculate the score, we considered the screen refresh rate (*N* = 75 Hz) and the software counted the number of times *n* (out of *N* screen updates) in which

(3) $$\begin{array}{l} |{\text{Target}}_{\text{center}}-\text{Cursor}{{\;}_{\text{center}}|}_{2}\\ \;<{\text{Target}}_{\text{radius}}+{\text{Cursor}}_{\text{radius}} \end{array}$$

where |.|_2_ denotes the Euclidean distance. The score in each trial was $\frac{n}{N}\times 100$. The last cursor position of the hold period, together with the target, was still visible on screen during presentation of the performance score, even in conditions that withheld visual feedback of the cursor during movement. Recording, online-processing, experimental control and user interface were handled by Python-based software, developed for these and similar experiments.

#### Experiment 1

Experiment 1 consisted of 480 trials, divided into two parts: a familiarization phase of 120 trials during which subjects received visual and artificial proprioceptive feedback (VF+PF condition) and a test phase of 360 trials with half of the trials running in VF+PF condition. The remaining trials were equally divided between conditions of only PF, without a visible cursor (PF condition), only visual feedback (VF condition) or neither of both kinds of sensory feedback (noFB condition). During the test phase different conditions appeared in a pseudorandom order so that in each set of 24 consecutive trials, each of the feedback conditions PF, VF and noFB were presented in combination with each of four targets exactly once, while in the same set of trials, condition VF+PF was combined with each target three times. Cursor position was controlled by muscles APB (DoA 135°, up left) and ADM (DoA: 45°, up right), as illustrated in Fig. 2a. All subjects in this experiment were naïve to the concepts of myoelectric control as well as PF.

#### Experiment 2

Experiment 2 consisted of 240 trials. During the first 120 trials, i.e. the familiarization phase, subjects received only visual feedback (VF condition). The test phase was equivalent to that of Experiment 1, with half of the trials running in VF+PF condition. The test phase consisted of only 120 trials. DoAs and controlling muscles were the same as in Experiment 1. This experiment was carried out with a new group of volunteers, who had not experienced PF before, that is, who did not participate in Experiment 1. They received their first experience of PF at the beginning of the test phase.

#### Experiment 3

Experiment 3 followed immediately after Experiment 2 with the same participants. To reduce the effect of prior training, for the new experiment, instead of APB and ADM, two previously unused muscles, 1DI and ECR, were used for cursor control. Experiment 3 consisted of 240 trials including a familiarization phase of 120 trials. The experimental conditions reflected those of Experiment 1 with the critical difference that during the PF and VF+PF condition, PF was not congruent to the cursor movement, but mirrored at the vertical midline so that the manipulandum guided the participant’s right hand to the left when the cursor moved the right and vice versa.

### E Performance Metrics

In order to evaluate overall task performance and to track learning, we calculated the Euclidean distance between the centers of cursor position P_cursor_ and target position P_target_ and averaged this over the duration of the hold period in each trial. We refer to this measure as ‘target mismatch’, an error measure, normalized so that a value of 1.0 reflects the radius between starting point P_start_ and the quarter-circle of the targets, whereas values close to zero indicate accurate matching of the target with little error:

(4) $$\text{Target}\;\text{mismatch}=\frac{{\left|P_{\text{cursor}}-P_{\text{target}}\right|}_{2}}{{\left|P_{\text{start}}-P_{\text{target}}\right|}_{2}}.$$

**Fig. 3:**
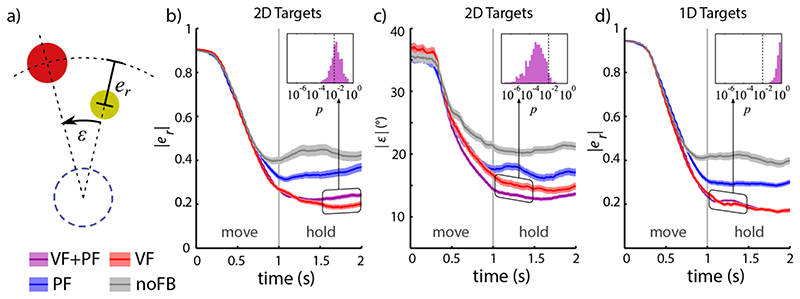
Average time-course of cursor trajectories over movement and hold period in Experiment 1. (a) Mismatch between cursor and 2D targets were separated into radial errors *e_r_* and angular errors *ε*. (b)-(d) Mean values over trials 121-480 from all subjects (coloured lines) ± 1 standard error (bands) are presented for different feedback conditions: VF+PF (purple), PF (blue), VF (red) and noFB (grey). (b) Absolute radial errors of target for 2D targets. Inset shows spread of *p*-values from 500 independent *t*-tests for differences between VF+PF (randomized sub-sample, cf. text) and VF in the time of 1.5 - 2.0 s - the dotted line represents *p* = 0.01. (c) Absolute angular errors for 2D targets (in degrees). Inset: spread of *p*-values for test VF+PF vs. VF, 1.0 - 1.5 s. (d) Absolute radial errors for 1D targets (1D movements do not allow for angular errors). Inset: spread of *p*-values for test VF+PF vs. VF, 1.0 - 1.5 s.

We further distinguished between the distance the cursor travelled and its direction from the starting point compared to target distance and direction, respectively, by converting cursor position to polar coordinates with the starting point as the origin. We defined absolute radial error |*ε*_r_|, indicating a mismatch in the magnitude of muscle contraction, and absolute angular error |*ε*|, reflecting errors in the relation between the activities of two muscles, illustrated in Fig. 3a. For the peripheral 1D targets, where control had only a single degree of freedom, no angular errors existed and radial errors were identical to the target mismatch measure.

### F Statistical Analysis

In several cases we compared two groups of samples and tested for significant differences in their means, using Student’s *t*-test for unpaired samples. When a family of comparisons was made, significance levels were adjusted to yield a family-wise error rate < 0.05 (Bonferroni correction). Before the *t*-tests, the normality of the data points was ascertained with a Shapiro–Wilk test.

## III Results

Our analysis focused on analyzing the subject’s ability to learn the presence and absence of the visual feedback condition. To avoid a bias that could be introduced because of non-learning subjects, we excluded those who could not gain viable control over the task from analysis. As a common criterion for the exclusion of subjects, we based this decision on trials 121 to 240, which had comparable conditions in all three experiments. Subjects were considered as non-learners, if the average target mismatch (Eq. 3) of all trials with visual feedback (conditions VF+PF and VF) was greater than 0.8. Thus, two subjects were excluded from Experiment 1, three from Experiment 2 and one from Experiment 3. Preliminary results of this work were published in [23].

### A Experiment 1

#### Within-trial dynamics

We examined task-related errors in the test phase of Experiment 1 as they evolved over movement and hold period. To separate specific features of myoelectric control, we distinguished between radial and angular errors (Fig. 3). On a grand average, errors were stationary over the time of the hold period, but displayed some notable differences in dynamics between feedback conditions. Differences between feedback conditions were found and compared within three separate time windows: the late movement period (0.5-1.0 s after target appearance), the early hold phase (1.0-1.5 s) and the late hold phase (1.5-2.0 s). Within the early movement period (0-0.5 s after target appearance), comprising reaction time and initial muscle activation before feedback correction, no significant differences occurred between conditions (Fig. 3b-d).

We used a *t*-test to determine whether the time-averaged differences between cursor trajectories of two conditions within a time window were significant. To calculate these differential measures, we paired up trials from the same subject, to the same target and from within the same 24-trial time frame of the experiment. Since VF+PF condition had three times more trials than the other conditions, only one matching VF+PF trial out of every three was randomly selected for a paired *t*-test. This random selection was repeated 500 times independently. Absolute angular errors for 2D targets decreased by ~5% in the VF+PF condition compared to the VF only condition during 1.5 – 2.0 s into the trial (Fig. 3c). However, this improvement was not observed in absolute radial errors. Insets in Fig. 3b-d show distribution of *p*-value for *t*-tests between VF+PF and VF conditions. A complete overview of all significant differences between feedback conditions is given in Fig. S1 (Supplementary Material).

#### Overall task performance

**Fig. 4:**
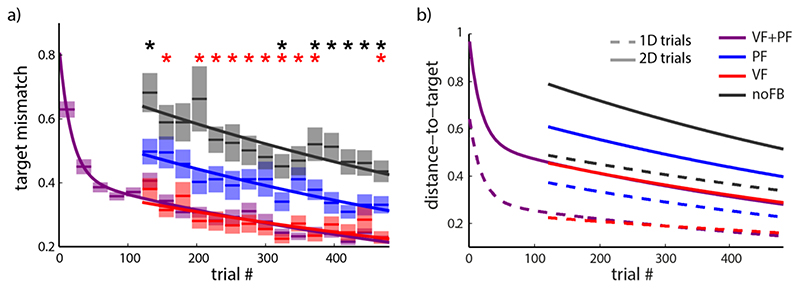
Learning of myoelectric-control in Experiment 1. (a) Target mismatch during the hold period, averaged over trials of the same condition within each set of 24 consecutive trials in the experiment. Data is pooled over all subjects. Semi-transparent boxes show ± standard error of the mean with mean indicated by solid lines in the middle. Overlaid are exponential fits for conditions PF (blue), VF (red) and noFB (black) and a double exponential fit for condition VF+PF (purple), which was the only condition during the initial learning phase. Paired *t*-tests were run between the PF trials and the VF or noFB trials. Red and black asterisks indicate significantly lower target mismatch in VF or significantly higher values in noFB condition, respectively, when compared to the PF condition. (b) Learning of myoelectric-control in Experiment 1, separated for 1D (dashed lines) and 2D trials (solid lines).

To evaluate overall task performance we calculated average target mismatch during the hold phase over a series of time windows to produce learning curves. Trials from all learning subjects were pooled and averaged over time frames corresponding to 24 trials but separating trials from different conditions within that period. Figure 4a reflects improvements of task performance in Experiment 1. Parametric fits are overlaid as solid lines. Temporal evolution of target mismatch in conditions PF, VF and noFB, that were only encountered in the test phase, could be well approximated by a single exponential fit (fit significance *p* < 0.05 for all fits with varying *r* ^2^), the more rapid initial learning phase in VF+PF condition was accounted for by the use of a double exponential function.

Figure 4 suggests that the presence of visual feedback allowed subjects to match the target during the hold period, irrespective of whether additional proprioceptive feedback was supplied (VF+PF condition) or not (VF condition). For this measure, no improvement over VF could be found in the VF+PF condition (multiple paired *t*-tests, Bonferroni corrected, *p* > 0.05).

A significantly lower average target mismatch in PF vs. noFB condition (Fig. 4a, black asterisks) and significantly higher average vs. the VF condition (red asterisks) was confirmed by applying a series of paired *t*-test over short stretches of 24 trials, represented by the averages shown in Fig. 4a, comprising one trial to each target for each of the three conditions (PF, VF and noFB) of each subject. Trials of different conditions were paired for the same target of the same subject. We used a Bonferroni correction for multiple comparisons (multiple *t*-test with post-hoc analysis), testing for a family-wise error rate of smaller than 0.05 (corresponding to *p* < 0.0033 for each single test). Therefore, task performance with PF as the only source of sensory feedback (PF) was consistently better than without feedback (noFB), but weaker than in VF or VF+PF condition. These differences were maintained throughout the course of learning.

Control errors for movements to the peripheral 1D targets (Fig. 4b) were lower than for the substantially more difficult case of two-dimensional control (2D targets). However, the relations between different feedback conditions were independent from target positions or dimensions.

Similar learning curves could be obtained using the score, presented to subjects at the end of each trial, as a performance metric (Fig. S2, Supplementary Material). However, although this measure was provided to the subject during the experiments as a simple and intuitive performance indicator, it is not as sensitive as the target mismatch index in distinguishing the differences between the four feedback conditions. The main reason behind this shortcoming is that, score introduces floor and ceiling effect, i.e. a cut-off at both scores 0 and 100 of the scale where no further distinction of performance is possible.

### B Experiment 2

With a new group of subjects, we tested whether subjects, in Experiment 1, showed higher performance in the early PF-alone trials because they have an innate mapping between visual and proprioceptive feedbacks or they acquired this mapping because they experienced these two feedbacks simultaneously during the initial familiarization block (VF+PF condition). Therefore, in Experiment 2, condition VF+PF in the familiarization phase of Experiment 1 was replaced with condition VF only, withholding any experience of PF until the onset of the test phase in trials 121-240.

**Fig. 5:**
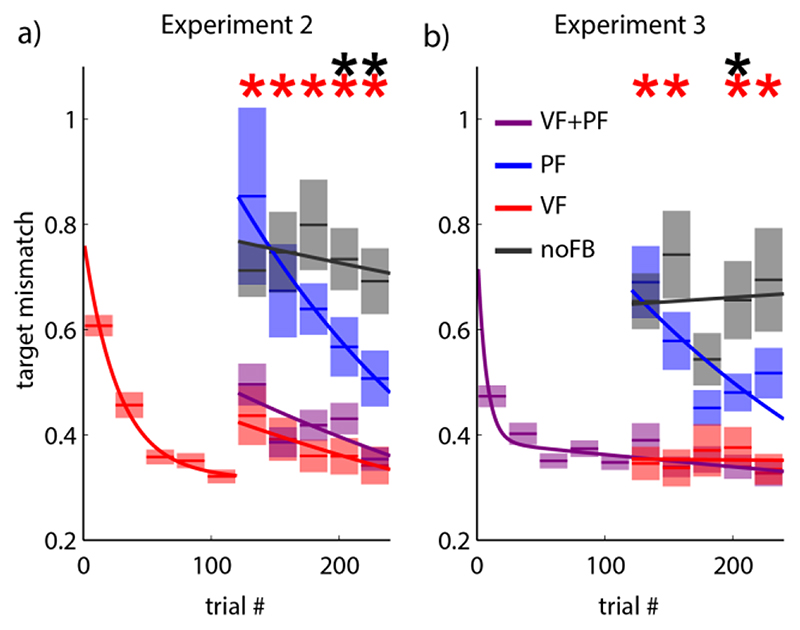
Experiments 2 and 3. (a) Experiment 2: familiarization phase with VF condition (instead of PF) followed by test sessions of VF, PF, VF+PF and no FB. b) Experiment 3: artificial proprioceptive feedback mirrored at vertical midline. Paired *t*-tests were run between PF trials and VF or noFB trials within a set. Red and black asterisks indicate significantly lower target mismatch in VF or significantly higher values in noFB condition, respectively, with a family-wise error rate ≤ 0.05 (Bonferroni correction).

While average target mismatch in VF+PF condition was still not significantly different from VF condition at the same stage of learning (multiple paired *t*-tests, Bonferroni corrected, *p* > 0.05), performance in PF condition equaled that of the noFB condition at the beginning of the test phase and only became significantly better in later trials (Fig. 5a) (multiple paired *t*-tests, Bonferroni corrected, *p* < 0.05, black asterisks). This indicates that artificial PF needs significant prior experience to be used as a source of feedback associated with the task, and that this association was in fact formed during the familiarization phase in Experiment 1. The learning curve for the VF condition shows a step towards higher control errors at the beginning of the test phase, when PF was first introduced as a new sensory modality to be processed. The second part of the curve in Fig. 5a was therefore fitted with a separate exponential function to accommodate this sudden change.

### C Experiment 3

With Experiment 3, we tested whether the integration of artificial PF into sensorimotor control was enabled by the fact that vision and artificial proprioception provided congruent feedback, or whether more arbitrary relations – specifically, with proprioception as a mirror image of vision – could be learned equally well. Therefore, in contrast to Experiment 1, we designed the handle movement providing artificial PF in Experiment 3 to be mirrored at the vertical midline.

The subjects started this experiment with a familiarization phase with both VF and PF. During the familiarization phase very rapid initial learning could be observed (Fig. 5b). This does not come unexpected, since subjects were not naïve to myoelectric control any more, after participating in Experiment 2. However, since a different set of EMGs was used, specifics of the control had to be trained anew.

Next when one or both of the feedbacks were removed, control errors were high for both PF and noFB conditions in the early trials of the test phase, indicating that integration of incongruent artificial PF into myoelectric control had been delayed. The red asterisks in Fig 5b indicate statistically meaningful differences between the PF and VF conditions. In later trials, accuracy in PF condition improved to significantly outperform condition noFB (black asterisk) (multiple paired *t*-tests with a family-wise error rate ≤ 0.05 - Bonferroni corrected) demonstrating that subjects could learn to use the mirrored feedback but only when the absence of visual feedback made the use of the mirrored feedback necessary for performance of the task.

## IV Concluding Remarks

Our findings demonstrate that the artificial proprioceptive feedback, supplied to the contralateral arm, improves myoelectric control in the absence of visual feedback. When visual feedback was available, overall performance was unaffected by the additional feedback, despite a small but significant reduction in errors in movement direction. This observation is compatible with previous reports that found that proprioceptive information was more effective to estimate direction than distance [18], [24]. This selective advantage of added artificial proprioceptive feedback emerged early in the movement, but was diminished towards the end of the hold period. We speculate both visual and proprioceptive feedback contribute during movement, whereas visual feedback, which allows for direct matching of visual cursor and target, takes precedence over proprioception control during the hold period. Direction errors must be controlled early to avoid large corrections later in the movement, thus artificial proprioceptive feedback may have a significant impact during this period. By contrast, errors in distance are only relevant towards the end of the movement and during the hold phase, when vision may be more advantageous than proprioception.

Based on studies of limb position drift during repetitive movements without visual feedback, Brown *et al*. in [26] concluded that separate controllers exist for limb position and movement. According to this hypothesis, position control relies more heavily on vision, while proprioception effectively informs movement control, such that the direction and length of individual movements remained constant as the drift of hand position accumulated. The differential impact of artificial proprioceptive feedback on movement and hold periods in our task adds further support to the notion that proprioceptive feedback may have a higher importance for movement than position control.

There were two reasons behind choosing muscles of the hand, instead of the forearm. First, we were interested in quantifying capability of intrinsic hand muscles in controlling myoelectric interfaces because, in the long-term, these results could contribute to design of biomimetic and abstract controllers for partial hand prostheses [20]–[22], [25]. As we discussed in [20], we avoided the intuitive DoAs to slow down the learning process and better observe improvements over time. In prosthetic applications the choice control signals may be restricted because of amputation, precluding intuitive control. These cases are, to some degree, better emulated by a non-intuitive design, such as ours. Previously, we showed that muscles of the forearm and hand show very similar tuning activity patterns when controlling a myoelectric interface [20]. Therefore, had we carried out Experiment 1 with the muscles of the left arm, it is likely that we would have observed very similar results.

Our second rationale was that the control of a myoelectric interface with the left hand muscles and receiving artificial feedback from the contralateral arm not only allows to test for distributed sensorimotor integration but also it imposes a secondary level of abstractness in feedback, that is proprioceptive information about activity of hand muscles are relayed back the brain via arm. Importantly, we showed that in this myoelectric interface design, proprioceptive feedback from the controlling muscles (here on the left hand) is very limited [21],[22].

In the current study, the EMG signal was used to control the position of the cursor. This design was consistent with our previous work on myoelectric controlled interfaces and helped with the interpretation of the results [20]–[22]. Further studies are required to determine whether other states such as cursor velocity or acceleration are better controllable through EMG. We detailed robot performance characteristic in Table I to highlight the technical constraints of our experiment and setup. We did not observe any performance effects that we could relate directly to the robot performance but cannot state with certainty that better robot performance will not improve the results.

Another important factor that will be considered is the time lag between the cursor (output of the motor system) and the robot position (sensory input) and its effect on sensorimotor integration. However, the investigation of the delay related effects was beyond the scope of the current work. In the current study we opted to keep the time lag between the cursor and the robot position as small as possible (Table 1).

In our first experiment, following an initial familiarization phase in which APF was supplied together with visual information (VF+PF), proprioception improved control as soon as visual feedback was withheld (test phase, PF condition). However, this did not occur if the familiarization phase consisted solely of visual feedback trials (Experiment 2). This is in agreement with finding of Mon-Williams *et al*. [16] who showed that when vision is available, in perceiving limb position, subjects trust the visual feedback more than what they feel through their touch sensory system. Together, they imply that sensory integration of the proprioceptive modality occurred implicitly during the familiarization phase, even though this additional feedback did not measurably impact task success when visual feedback was available.

In Experiment 2, we tested whether the higher performance in early PF-alone trials in Experiment 1 was because subjects had an inherent mapping between congruent visual and proprioceptive feedbacks or they acquired this mapping because they experienced these two feedbacks simultaneously during the initial familiarization block. Results showed that if the familiarization block contained only VF trials, the performance in the early PF-alone trials are as low as that in no-FB trials. This finding supports strongly the notion of multi-sensory integration during the familiarization block when these two feedbacks are presented simultaneously. The sudden deterioration in task-related performance in the VF condition, after introducing three new feedback conditions, i.e. VF+PF, PF and noFB, to the task at the beginning of the test phase may have resulted because the relative weightings of the visual and proprioceptive sensory feedback modalities must be updated in the motor program for all conditions in parallel and within a short space of time. A potential mechanism to adjust the weights could be to decrease the weighting of visual feedback and increase incrementally the weighting of the proprioceptive feedback. This approach consequently leads to performance degradation in the VF-only condition. An alternative, but simpler, explanation could be that the performance drop in VF-only condition is the result of a general increase in computational load required to integrate the additional feedback modality into myoelectric control. Further work will be required to determine how multi-sensory integration and learning evolves in unfamiliar and abstract tasks such the one we proposed in this article.

In Experiment 3, we tested whether this implicit integration of artificial proprioception into sensorimotor control required congruent visual and proprioceptive information. We found that unlike in the case of congruent feedback, proprioceptive feedback was not incorporated implicitly into the control strategy despite its provision with visual feedback during the familiarization phase. This is consistent with the findings of [28] that proprioception is not used for sensorimotor adaptation, when observed motor errors conflict with vision. However, even an incongruent proprioceptive feedback could improve performance in the absence of visual feedback. This was only seen late in the test phase after training on this specific condition (PF condition). Therefore, we believe that proprioceptive information is weighted less during the learning process when it contradicts, or at least when does not fully agree with, visual feedback.

Control of a dexterous hand prosthesis in an unpredictable environment will benefit from the provision of fast, reliable and potentially multi-modal sensory feedback [29], [30]. Non-visual feedback modalities can aid myoelectric control when vision is unavailable, and may help the prosthesis become incorporated into the wearer’s body image [31]. For the foreseeable future, the only way to deliver proprioceptive feedback of the posture or position of a prosthesis is through intact sensory pathways. Previous efforts have focused on substituting other sensory modalities, such as non-invasive tactile stimulation [2],[3] or via the use of the invasive targeted sensory re-innervation [32]. Our results suggest an alternative strategy may be to substitute the proprioceptive faculties of another limb. In either case, finding appropriate targets for effective proprioceptive feedback remains a challenge, because the artificial sense must not hinder the normal function of intact pathways. Nevertheless, the flexibility of the motor system to incorporate proprioceptive information from other limbs presents opportunities to improve the usability of prostheses by exploiting the range of alternative sensory channels available to amputees [33] The proposed paradigm, in this current design, may not be useful in real-life prosthetic control applications since it may not be practical to provide proprioceptive feedback in the healthy arm. Our motivation is to possibly provide the proprioception corresponding to a prosthesis to one of the finger (possibly the little finger which is used least) of the healthy arm.

The proposed task structure can provide a flexible paradigm by which the effectiveness multi-sensory integration in different feedback conditions can be evaluated.

## Supplementary Material

Supplementary material

## Figures and Tables

**Table I:** Accuracy of manipulandum

PF+VF (PF)	1D targets		2D targets	
	Movement phase	Hold phase	Movement phase	Hold phase
r_x_	0.93 (0.95)	0.72 (0.69)	0.74 (0.74)	0.77 (0.75)
r_y_	0.93 (0.95)	0.72 (0.68)	0.96 (0.95)	0.67 (0.66)
*lag*	0 (0)	6 (6)	0 (0)	17(13)
*d*(cm)	1.3 (1.2)	1.9 (1.6)	1.6 (1.5)	2.7 (2.3)

Accuracy of manipulandum matching the cursor position in PF+VF condition or the hypothetical cursor position in PF condition (in brackets). *r_x_/r_y_*: correlation coefficients of trajectories in horizontal and vertical direction, respectively; average over trials. *lag* (ms): median time lag between the visual cursor and the manipulandum (lag of maximum cross covariance). *d* (cm): average distance between cursor and manipulandum.
