# Supplementary material for: Artificial Proprioceptive Feedback for Myoelectric Control
Source: IEEE Trans Neural Syst Rehabil Eng. Author manuscript; Available in PMC 2021 Jun 14. (PMC7610977; doi:10.1109/TNSRE.2014.2355856)
Supplement: Supplementary material [file EMS126972-supplement-Supplementary_material.pdf]

Supplementary Figures

We propose to include two supplementary figures.

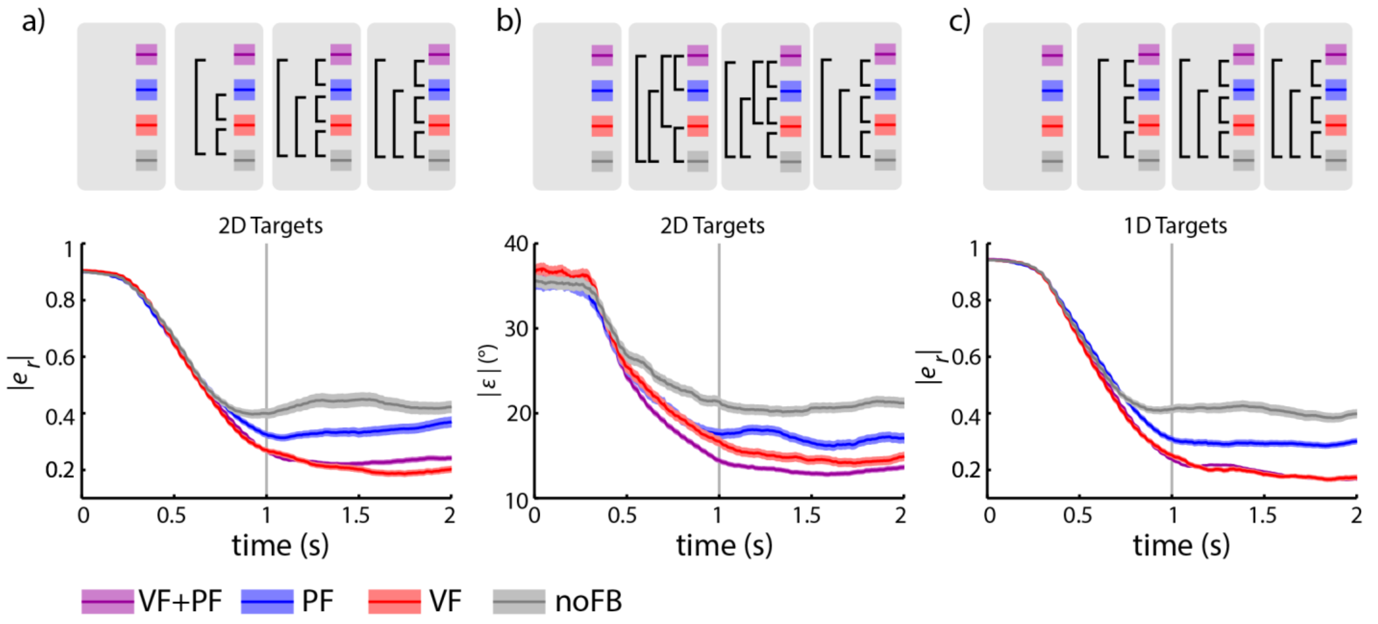

Fig. S1. Significant differences between conditions over 4 time periods in the trials (0 – 0.5 s; 0.5 – 1.0 s; 1.0 – 1.5 s; 1.5 – 2.0 s). The top row identifies conditions with significant differences (illustrated with brackets, paired *t*-test, corrected for multiple paired *t*-test comparisons, $p < 0.01$) in the respective measure within the corresponding time period. (a) Radial errors for 2D targets. (b) Angular errors for 2D targets. (c) Radial errors for 1D targets. Graphs in the second row are the same as in Fig. 3b-d

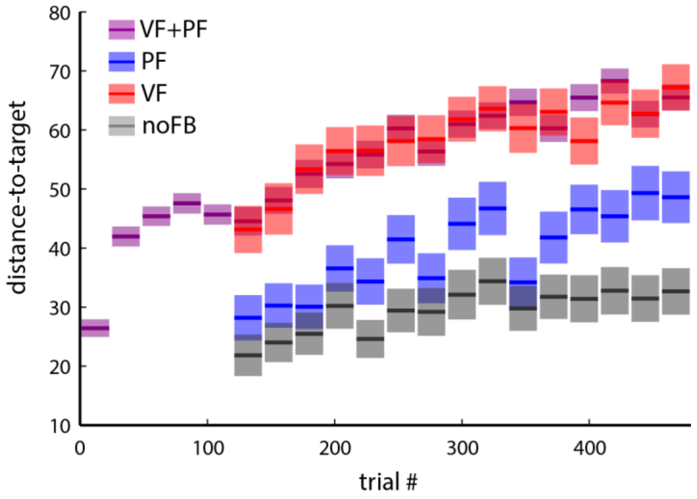

Fig. S2. Task performance in terms of the average score in Experiment 1, as presented to subjects. Scores reflected the percentage of time the cursor overlapped with the target during the one second hold phase. Note that differences in low average values become in-discriminable since they are dominated by zero scores.
